# Thermo-mechanical characterization of electrospun polyurethane/carbon-nanotubes nanofibers: a comparative study

**DOI:** 10.1038/s41598-023-44020-x

**Published:** 2023-10-13

**Authors:** A. Shaker, Amira T. Khedewy, Mohamed A. Hassan, Marwa A. Abd El-Baky

**Affiliations:** Mechanical Design and Production Engineering Department, Zagazig University, Zagazig, 44519 Egypt

**Keywords:** Nanoscale materials, Soft materials, Structural materials, Mechanical engineering

## Abstract

Creating ultrathin, mountable fibers from a wide range of polymeric functional materials has made electrospinning an adequate approach to producing highly flexible and elastic materials. In this paper, electrospinning was utilized to produce thermoplastic polyurethane (TPU) nanofibrous membranes for the purpose of studying their thermal and mechanical properties. Towards a study of the effects of fiber orientation and multi-walled carbon nanotubes (MWCNTs) as a filler on both mechanical and thermal characteristics of electrospun TPU mats, an experimental comparison was held between unidirectional and randomly aligned TPU and TPU/MWCNTs nanofibrous structures. The incorporation of MWCNTs into randomly oriented TPU nanofibers resulted in a significant increase in Young's modulus (E), from 3.9 to 7.5 MPa. On the other hand, for unidirectionally spun fibers, Young's modulus increased from 17.1 to 18.4 MPa upon the addition of MWCNTs. However, dynamic mechanical analysis revealed a different behavior. The randomly oriented specimens exhibited a storage modulus with a significant increase from 180 to 614 MPa for TPU and TPU/MWCNTs mats, respectively, and a slight increase from 119 to 143 MPa for unidirectional TPU and TPU/MWCNTs mats, respectively. Meanwhile, the loss modulus increased with the addition of MWCNTs from 15.7 to 58.9 MPa and from 6.4 to 12 MPa for the random and aligned fibers, respectively. The glass transition values for all the mats fell in the temperature range of – 60 to − 20 °C. The thermal degradation of the membranes was not significantly affected by the addition of MWCNTs, indicating that the mixing of the two constituents did not change the TPU’s polymer structure and that the TPU/MWCNTs nanocomposite exhibited stable thermal degradation properties.

## Introduction

The demand for lightweight yet strong structures in various applications has led to the exploration of novel designs using polymeric nanofibers^1^. Nanofibrous membranes with enhanced thermomechanical properties have garnered considerable interest in fields such as energy storage^2^, filtration^3^, tissue engineering^4^, and stretchable electromechanical devices^5^. Electrospinning is a widely employed manufacturing technique used to produce thin, non-woven nanofibrous films by applying high voltage between conductive electrodes, resulting in fibers with diameters typically in the hundreds of nanometers^6,7^. This technique has gained widespread attention due to its high efficiency, simplicity, and ability to spin inorganic materials^8^.

Although electrospinning is commonly used for spinning nanofibers from polymers, recent studies suggest that it is also possible to electrospin other materials, such as carbon-based materials and ceramics^9^. Nevertheless, polymers remain the predominant choice for electrospinning due to their unique characteristics and versatility. Polymers offer a wide range of tunable properties, including mechanical strength, flexibility, biocompatibility, and chemical stability, making them well-suited for various applications^10^. Polymers most commonly used in electrospinning include poly(lactic acid) (PLA)^11^, poly(caprolactone) (PCL)^12^, poly(vinyl alcohol) (PVA)^13^, poly(ethylene oxide) (PEO)^14^, and thermoplastic polyurethane (TPU)^15^. TPU is a highly versatile material widely used in various industrial sectors, such as biomedical, electronics, and automotive engineering, owing to its exceptional mechanical properties, including low compression set, high resilience, and resistance to tears, abrasions, impacts, and environmental factors^16,17^.

In recent years, the effect of fiber orientation on the properties of electrospun membranes, particularly their mechanical behavior, has grabbed the attention of researchers. Kijeńska-Gawrońska et al.^18^ investigated the combined effect of fiber diameter and orientation on the mechanical and thermal properties of TPU electrospun membranes and observed that while fiber orientation had a significant effect on the mechanical properties, the impact of fiber diameter was minimal. Furthermore, both parameters had little influence on the thermal characteristics of the mats. Pham Le et al.^19^ explored the mechanical properties of polyvinyl chloride (PVC) nanofibers, where different speeds of a rotating drum were used to obtain varying fiber orientation angles. The study revealed that higher rotating speeds resulted in closer orientation angles approaching 0° and the Young's modulus (E) was significantly affected by the fiber orientation, with E for membranes with fibers aligned at 0° being twice that of randomly oriented fibrous membranes. Similar findings were reported by Maciel et al.^20^.

Another large area of research that is gaining recognition in the electrospinning field is the incorporation of various nanomaterials in the electrospun membrane. The resulting composite nanofibers could possess improved electrical^21^, magnetoelectric^20^, photosensitive^22^, thermal^23^, and antibacterial^24^ properties, thus making them suitable for use in a much broader spectrum of fields^25^. These nanocomposites have a wide range of possible applications, including but not limited to sensing devices^26^, wearable electronics^27^, drug-delivery agents, antibacterial fields^28^, and anti-static coatings^29^. The nano-additives can be made of materials ranging from metals to metal oxides, carbon, and polymers^30^. Carbon nanotubes (CNTs) as a filler material are interesting due to their capacity to enhance the thermal behavior, mechanical properties, and electrical conductivity of polymer nanocomposites, even at very low weight percentages of the polymer^31^.

Eivazi et al. ^32^ studied the influence of varying concentrations of CNTs in randomly oriented TPU electrospun nanofibers for biomedical applications, ranging from 0.01 to 1 wt.%. The results demonstrated that the inclusion of CNTs led to notable improvements in the Young's modulus, conductivity and toughness, however, it was observed that the maximum strain of the nanofibrous mats experienced a noticeable decrease as a consequence of increasing the CNTs percentage. Similar results were reported by Özkan et al.^33^, who compared the mechanical properties of TPU, TPU/CNTs, and TPU/graphene randomly aligned nanofibrous membranes. The inclusion of CNTs increased the tensile strength of the materials, while graphene had a significant detrimental effect on the mechanical characteristics.

Recent studies show a promising future in stretchable electromechanical devices for TPU/CNTs nanofibrous materials. Wang et al. ^34^ manufactured MWCNTs/TPU nanofiber films that were flexible, had a wide temperature range, and improved combustion carbon performance. Tang et al.^35^ developed a highly-stretchable CNTs/TPU composite nanofiber yarn for the development of strain sensors for smart sports bandages. Huang et al.^36^ developed a highly stretchable, conductive, and sensitive strain-sensing material based on a CNTs-bridged AgNPs strain sensor and had promising applications in flexible and wearable devices. Han et al.^37^ developed multi-responsive actuators based on modified electrospun films with MWCNTs.

This study delves into the incorporation of multiwalled carbon nanotubes (MWCNTs) (denoted by CNT for more simplicity) into thermoplastic polyurethane (TPU) electrospun nanofibrous membranes. The membranes were manufactured in two different fiber orientations, namely, random and unidirectional alignment. The aim of the investigation was to examine the impact of CNT addition on the thermomechanical properties of the TPU membranes. However, given the limited existing research in this area, a major goal of this paper was to gain a deeper understanding of how fiber orientation influences the mechanical and thermal behaviors of these membranes. Such insights are crucial for optimizing the performance of these membranes in a wide range of future applications, including soft actuators and strain sensors.

## Experimental work

### Materials

The polymer being electrospun is thermoplastic polyurethane (TPU) S80A supplied by Elastollan Co., Ltd, Japan. N,N-Dimethylformamide (DMF 94% Alpha Chemika, India) was utilized as a solvent for TPU to prepare the polymer solution. Muti-walled carbon-nanotubes (MWCNTs) with an average diameter of 10 nm and an aspect ratio greater than 100 were purchased from Sigma–Aldrich Co., Ltd, USA, and used as the reinforcement phase in the composite.

### Electrospinning of TPU and TPU/CNT mats

To conduct a comprehensive and comparative investigation, four types of membranes were manufactured by electrospinning technique. Pure TPU and TPU/CNT solutions were prepared and electrospun into randomly oriented and unidirectionally aligned fiber membranes.

**Figure 1:**
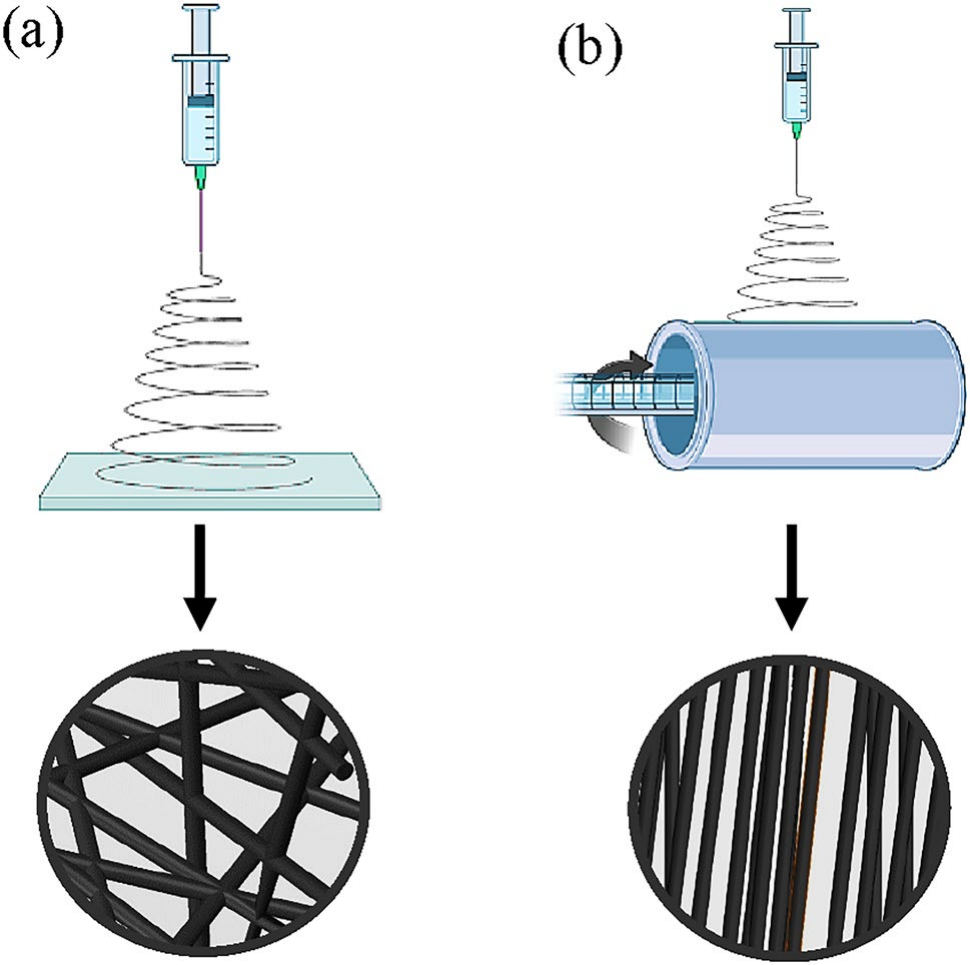
Electrospinning schematic diagrams for (**a**) random and (**b**) aligned nanofibers.

To prepare the TPU/CNT solution, TPU bullets were dispersed in DMF at a concentration of 15 wt.% and stirred for 8 h at room temperature. CNT were uniformly dispersed in the DMF at a concentration of 0.2 wt.% (1.3 wt.% of TPU) using an ultrasonic processor (HIELSCHER UP 200S, Germany) prior to the addition of the TPU bullets. The resulting solution was loaded into a 5 ml syringe and inserted into the NANON-01B electrospinning setup (MECC CO., LTD Japan). A dehumidification setup was connected to the electrospinning unit to ensure a controlled environment with minimal humidity during the manufacturing process to prevent the formation of beads. To fabricate the randomly oriented nanofibrous membranes, a flat collector was used to gather the electrospun fibers, while a rotating drum was employed to collect the unidirectional fibers for the other membranes as shown in Fig. 1.

To optimize the electrospinning process, various parameters such as applied voltage, feed rate, stand-off distance, spinneret speed, and humidity percentage are often adjusted^38^. After multiple iterations, the optimal parameters were determined for the electrospinning of both pure TPU and TPU/CNT solutions and utilized for membrane fabrication. A direct current voltage of 25 kV and a feed rate of 0.76 ml/h were used to fabricate randomly oriented and unidirectional pure TPU membranes, while TPU/CNT membranes were produced using a direct current voltage of 28 kV and a feed rate of 0.4 ml/h. A stand-off distance of 150 mm was maintained between the needle tip and the collector during all fabrication processes. The rotating drum speeds used for the fabrication process were 1500 and 1200 rpm for pure TPU and TPU/CNT membranes, respectively, which are equivalent to tangential speeds of 31.4 and 25.1 m/s, respectively. The electrospinning process was conducted at a relative humidity percentage of approximately 23 $\pm$ 3%, which was automatically maintained using the dehumidification unit. The fabricated membranes were finally laser-cut into standard dimensions for characterization using a CO_2_ laser machine (VLS 2.30; Versa Laser Systems, Scottsdale, AZ, USA) with a power of 2.0 watts, an optical focal length of 50.8 mm, and a pulse density of 393.7 pulse/in.

### Characterization

First, scanning electron microscopy (SEM) (JEOL JSM-6510 IV, Japan) was used to characterize the morphology of the electrospun membranes. Image-J software was employed to measure approximately 200 random fiber diameters, extracted from three distinct SEM images, for each membrane. The average fiber diameter of the mats was then analyzed and calculated based on the collected data. Transmission Electron Microscopy (TEM) observations were carried out by using HR-TEM (JEOL,JEM-2100,Japan). The composition and structure of the membranes were then analyzed using X-ray diffraction (XRD) spectrometry (X’Pert3 Powder, Malvern PANalytical, UK) in the range of 4° to 70° with a time step of 0.6 s, and Fourier transform infrared (FTIR) spectrometry (Nicolet iS10, ThermoFisher Scientific, UK) with a wave number range of 650–4000 cm^-1^.

**Figure 2:**
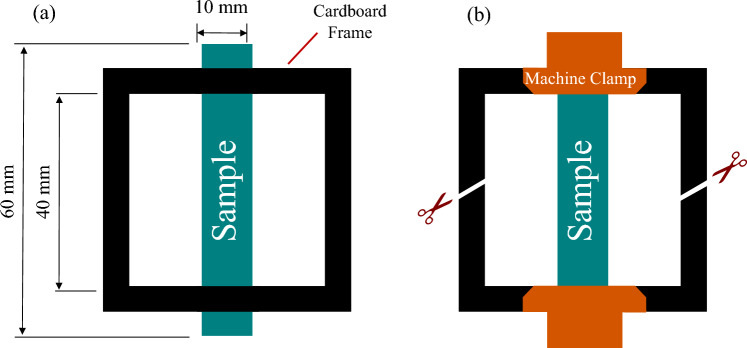
Preparation of tensile test samples; (**a**) test sample attached to the sample holder, (**b**) sample clamped onto the machine and cutting the sample holder sides.

The mechanical properties of the nanofibrous membranes were investigated through tensile and elastic recovery tests. Tensile testing was performed on a universal testing machine (Zwick Z010, Germany) with a crosshead speed of 50 mm/min and 100 N load cell. Three rectangular specimens (10 × 60 mm) were laser-cut and affixed onto a cardboard frame to facilitate secure fixation onto the machine grippers^39^. After clamping the specimen, the cardboard frame was cut to initiate the test, as shown in Fig. 2. Young’s modulus (E), maximum value of stress (σ_max_ ) and strain at maximum stress (ε_max_) were determined as the average value of the tested samples.

The tensile stress was calculated using two different methods: first, the standard mat cross-section normalization method (SN), which was proven to be unreliable for such nanofibrous mats and dependent on the method with which the membrane thickness was measured, which in this study’s case was a pneumatic digital micrometer. The second method is Mass-based normalization (MN), where the tensile stress was calculated using Eq. (1) ^40^,

1 $$\upsigma = {\uprho }_{m}\frac{F}{m}\mathrm{ L}$$

where m is the mass of the sample (measured in mg), ρ_m_ is the material density, F is the force (measured in N), L is the initial length of the sample (measured in mm) and σ is the stress expressed in MPa. The density corrections of the samples were performed according to the weight percentage of the nanoparticles^41^. This type of stress calculation enables more realistic values compared to the standard approach, where the cross-section area of the sample is used. Young’s modulus was calculated from the slope value of the stress–strain curve when the strain was 10%^42,43^.

Additionally, an elastic recovery test was conducted on the same setup under stress control with a crosshead speed of 100 mm/min. The hysteresis of elastic recovery was studied by applying 20 cycles of 50% of the maximum tensile stress obtained from the initial tensile test^44^. The thermal stability of the materials was evaluated using thermogravimetric analysis (TGA) (Mettler Toledo TA-TGA), where the samples were heated in an N_2_ atmosphere to 600 °C at a heating rate of 15 °C/min.

Lastly, a dynamic mechanical analysis (DMA) (Triton Instruments, Lincolnshire, UK) was conducted in tension mode to investigate the viscoelastic properties of the polymeric membranes. Rectangular samples (10 × 25 mm) were tested, and the temperature was controlled using liquid Nitrogen over a range of − 60 to 100 °C with an oscillation frequency of 1.0 Hz and a scanning rate of 5 °C/min. DMA is a powerful technique that can provide valuable insights into the viscoelastic behavior of materials, including their storage modulus, loss modulus, damping ratio, and response to mechanical stress as a function of multiple parameters, including temperature and frequency^45–47^. The combination of these techniques provided a comprehensive characterization of the electrospun membranes, enabling a better understanding of their thermo-mechanical properties and potential applications.

## Results and discussion

### Morphology

**Figure 3:**
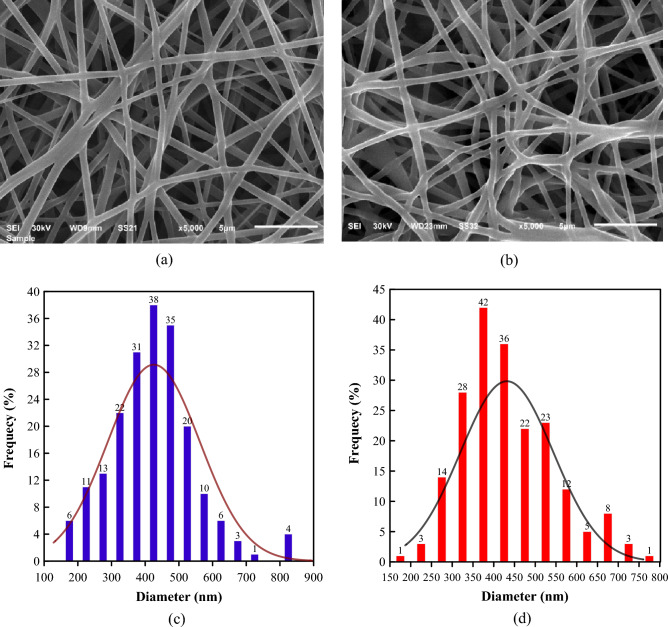
SEM images of randomly oriented (**a**) pure TPU and (**b**) TPU/CNT membranes, and fiber diameter distribution of randomly oriented (**c**) pure TPU and (**d**) TPU/CNT membranes.

Figures 3a and b show the SEM images of the randomly oriented pure TPU and TPU/CNT membranes, respectively. The images revealed clear, smooth 3-D fibrous structures in random orientations, with varying fiber diameters and an obvious absence of beads. Furthermore, Fig. 3c and d demonstrate the fiber diameter distribution for both the pure TPU and the TPU/CNT randomly oriented mats, respectively.

**Figure 4:**
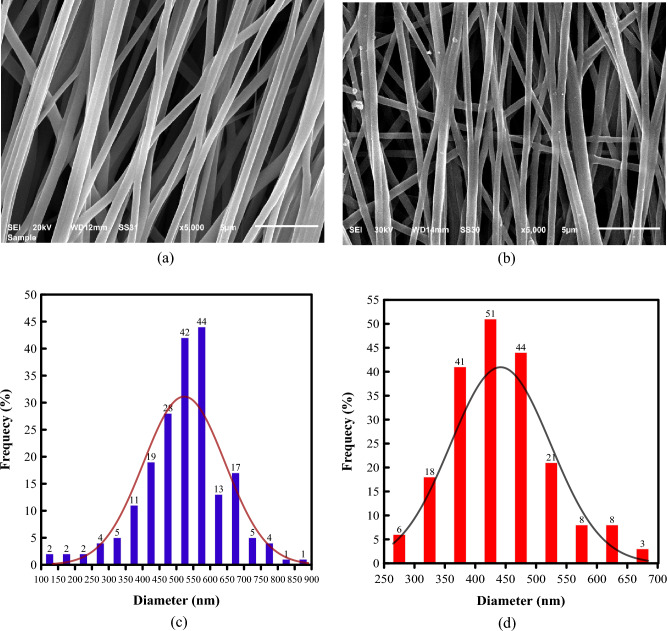
SEM images of unidirectionally aligned (**a**) pure TPU and (**b**) TPU/CNT membranes, and fiber diameter distribution of unidirectionally aligned (**c**) pure TPU and (**d**) TPU/CNT membranes.

The SEM images for the unidirectionally electrospun pure TPU and TPU/CNT membranes, however, are illustrated in Fig. 4a and b, respectively. While the fibers in the pure TPU sample show a slight deviation from the 0° spinning angle, most of the fibers have the same uniform orientation. On the other hand, the fibers in the TPU/CNT membranes have a more 0° spinning angle, but multiple fibers can be seen to have a completely different orientation. Figure 4c and d show the fiber diameter distribution for unidirectionally aligned pure TPU and TPU/CNT membranes, respectively. The average fiber diameter along with some statistical calculations of the fiber distribution for all four membranes are presented in Table 1.

**Table 1:** Fiber diameter analyses for TPU and TPU/CNT membranes.

Sample	Average Fiber Diameter (nm)		Max. Dia. (nm)	Min. Dia. (nm)	Mode	Variance
	$\varphi \pm SD$	CV%				
Unidirectional pure TPU	523 $\pm 121$	23	889	134	443	14,618
Unidirectional TPU/CNT	442 $\pm 84$	19	684	265	460	6832
Random pure TPU	426 $\pm 52$	12	1203	128	341	18,835
Random TPU/CNT	432 $\pm 112$	26	760	187	360	12,613

**Figure 5:**
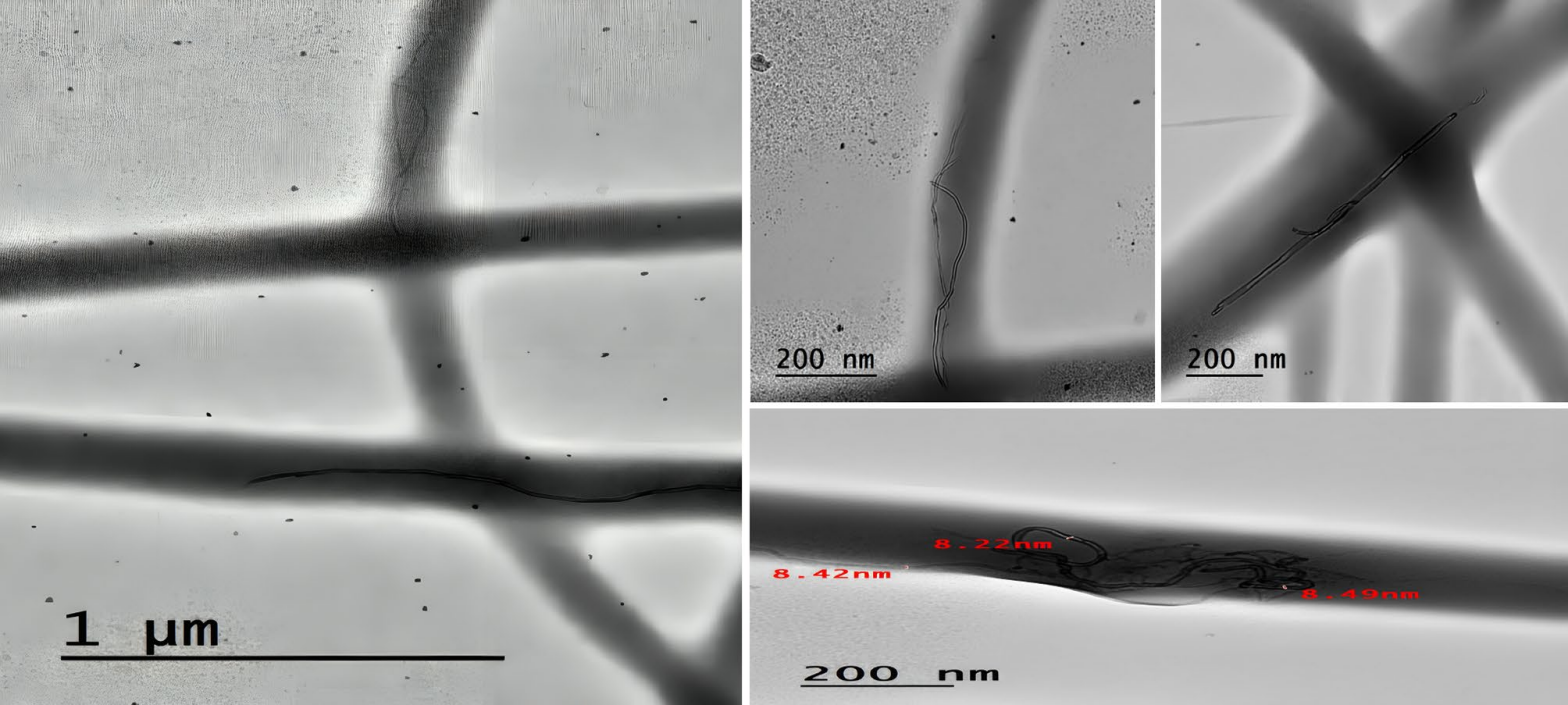
TEM images of electrospun TPU/CNT nanofibrous mat.

Transmission electron microscopy (TEM) is a valuable technique for investigating the disposition of CNT on electrospun nanofibers, primarily due to the higher density of CNT compared to the polymer matrix^48^. In TEM images, CNT with hollow structures appear as darker concentric tubular features embedded within the polymer nanofibers, in contrast to the uniform appearance of the fibers. Figure 5 displays TEM images of the TPU/CNT mats, with the CNT exhibiting a darker contrast compared to the TPU matrix, indicating their different densities. The images reveal a remarkable uniform distribution of CNT within the nanofibers, devoid of any noticeable aggregation. Notably, several CNT display an intriguing parallel alignment along the axis of the nanofiber, lending credence to the notion of an organized and well-dispersed arrangement. This observation offers compelling evidence of an aligned and evenly spread distribution of CNT within the electrospun nanofiber matrix. These findings align with similar studies conducted by other researchers^49,50^.

### XRD and FT-IR

**Figure 6:**
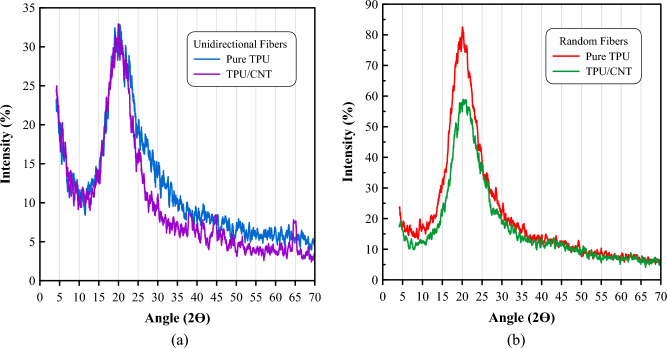
XRD spectra of (**a**) unidirectional pure TPU and TPU/CNT membranes, and (**b**) randomly oriented pure TPU and TPU/CNT membranes.

The structural transitions caused by interactions of the covalent bonds among the functional groups of the electrospun composite phases were studied using both XRD and FT-IR characterization techniques. Figure 6 shows the XRD patterns of TPU and TPU/CNT mats. The TPU nanofibrous membrane has a broad peak around 2Ө = 20°, while according to the literature, the MWCNTs have a diffraction peak at 2Ө = 26.03°^51,52^. The XRD pattern of TPU/CNT fibrous mat reveals one characteristic peak at 2Ө similar to that of TPU with downshifted intensity and more pronounced in randomly oriented mats, Fig. 5b, revealing that the inclusion of MWCNTs produced an orderly arrangement of TPU molecular chains^53^. The complete dispersion of CNT within the TPU matrix is indicated by the lack of a peak at 2θ = 26.03° in the TPU/CNT composite^51,52,54^.

**Figure 7:**
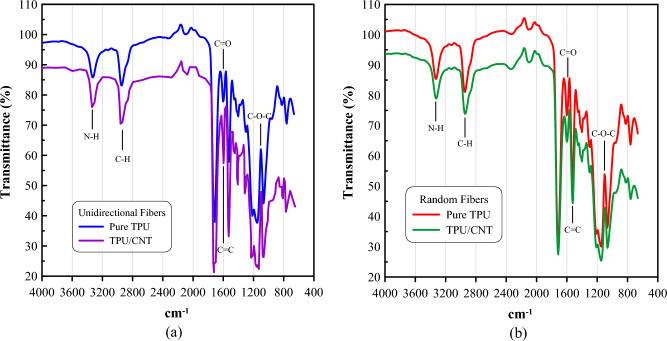
FT-IR spectra for (**a**) unidirectional pure TPU and TPU/CNT membranes, and (**b**) randomly oriented pure TPU and TPU/CNT membranes.

FT-IR spectra of TPU and TPU/CNT in both orientations are shown in Fig. 7. Typically, the spectrum of the TPU membrane shows peaks at 1070 cm^−1^ for (C–O), 1232 cm^−1^ for (C–C), 1569 cm^−1^ for (C=C), 1670 cm^−1^ for (C=O), 292 cm^−1^ for (C– H), and 3423 cm^−1^ for (N–H) ^39^, while for MWCNTs the spectrum shows peaks at 1097 cm^-1^ for (C–O), 1698 cm^-1^ is for (C=O), and 2924 cm^-1^ for (C–H) ^55^.

From the figure, it can be noted that all the materials show similar peaks at different intensities, and the intensity increases with an increase in polymeric chain length. The peaks shown in Fig. 7. are located at 1073 cm^−1^ (C–O), 1595 cm^−1^ (C=C), 1795 cm^−1^ (C=O), 2952 cm^−1^ (C–H), and 3325 cm^−1^ (N–H). The presence of peaks for (C=C) bonds is due to the addition of CNT. However, the addition of CNT did not result in any significant alterations to the chemical structure of the nanofibers in general^54,56,57^.

### Tensile and elastic recovery tests

**Figure 8:**
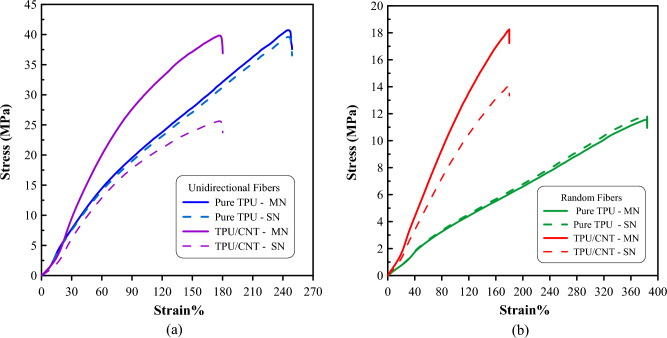
Stress–strain diagram for (**a**) unidirectional pure TPU and TPU/CNT membranes, and (**b**) randomly oriented pure TPU and TPU/CNT membranes.

**Figure 9:**
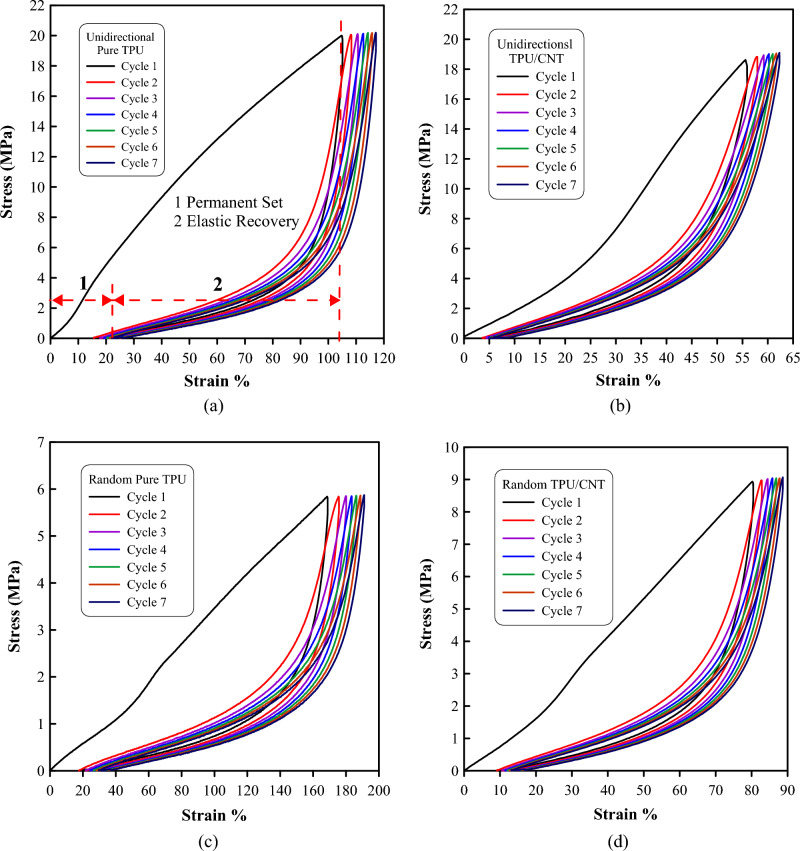
Cyclic stress–strain diagram for (**a**) unidirectional pure TPU membrane, (**b**) unidirectional TPU/CNT membrane, (**c**) randomly oriented pure TPU membrane, and (**d**) randomly oriented TPU/CNT membrane.

To investigate the mechanical properties of the electrospun nanofibrous mats and ensure high levels of stretchability and durability, tensile and elastic recovery tests were conducted. The recorded stress-strain and cyclic stress-strain curves are presented in Figs. 8 and 9, respectively. Figure 8 illustrates the tensile stress-strain relationships of each material, employing two distinct curves. The solid lines correspond to the mass-based normalization (MN) results, while the dashed lines represent the mat section normalization (SN) results.

The results indicate that while the pure TPU nanofibrous mats have significantly higher stretchability than the TPU/CNT sample for both fiber alignments, as shown in Fig. 8, the addition of CNT increases Young’s modulus for the nanofibrous mats. Figure 8a showcases that the presence of CNT in the TPU unidirectional membranes has increased Young's modulus by 7.6%, while reducing the tensile strength by 2.2% and the maximum strain by 33.6%. This behavior may be attributed to the interaction between TPU and CNT at an intermolecular level, which adversely affects the stretchability of electrospun TPU^58,59^. Furthermore, the TPU and TPU/CNT fibrous membranes in randomly oriented fiber alignment displayed similar behavior, with the exception of maximum stress value, as shown in Fig. 8b. In this case, the tensile strength of pure TPU has increased by 56.4% due to the presence of CNT content, and Young's modulus has been increased by 92.3%, while the maximum strain has been reduced by 48.8%. Table 2 provides a summary of the data of stress-strain curves for TPU and TPU/CNT membranes. Table 3 compares the mechanical properties of TPU and TPU/CNT mas obtained in this study with the characteristics of other electrospun nanofibrous mats found in literature.

**Table 2:** Stress–strain curves data for TPU and TPU/CNT membranes.

Sample	Young’s modulus (MPa)		Tensile strength (MPa)		Max. strain (%)	
	E $\pm SD$	C.V%	$\sigma \pm SD$	C.V%	ε $\pm SD$	C.V%
Unidirectional pure TPU	17.1 $\pm 2.5$	15%	40.7 $\pm 2.8$	7%	249 $\pm 10$	4%
Unidirectional TPU/CNT	$18.4\pm 1.7$	9%	39.8 $\pm 5.9$	15%	180 $\pm 30$	17%
Random pure TPU	3.9 $\pm 0.4$	11%	11.6 $\pm 1.3$	11%	384 $\pm 42$	11%
Random TPU/CNT	$7.5 \pm 0.9$	12%	18.3 $\pm 2.4$	13%	180 $\pm 25$	14%

**Table 3:** A comparison of the mechanical properties of the current study's nanofibrous mats with previous literature findings.

Work	Mat	Alignment	E (MPa)	${\varvec{\upsigma}}$_max_ (MPa)	ϵ_max_ %	Ref
Wijayanti et al. (2022)	PVA	Random	4.33	1.49	35.33	^60^
Pham Le et al. (2021)	PVC	Random	53	2.2	26	^19^
		Uniform	308	9.1	30	
Bazbouz et al. (2010)	Nylon6	Random	19.4	10.45	250	^61^
Huang et al. (2021)	PTFE	Random	2	2.5	123	^62^
Zaarour et al. (2019)	PVDF	Uniform	82.7	10.8	48.8	^63^
Sathirapongsasuti et al. (2021)	PVDF	Random	143	18	61	^64^
	PBS	Random	193	24	58	
Ahmadi et al. (2020)	PAN	Random	58.9	2.58	39.5	^56^
	PAN/CNT	Random	49.39	2.19	67.56	
Current Work	TPU	Random	3.9	11.6	384	–
		Uniform	17.1	40.7	249	–
	TPU/CNT	Random	7.5	18.3	180	–
		Uniform	18.4	39.8	180	–

In Fig. 9, the elastic recovery behavior of TPU and TPU/CNT nanofibrous membranes with random and unidirectional fiber orientations is compared. The figures show the first seven cycles of a twenty-cycle elastic recovery behavior test, where significant hysteresis was observed with distinct loading and unloading paths for each cycle. Upon reloading, all four mats exhibited increased compliance, indicating stress softening behavior with higher strain during the second loading curve. The reloading curves shifted towards higher strains than the preceding loading cycle, indicating permanent set. The elastic recovery was quantified by comparing the difference between applied strain and permanent set for each loading–unloading cycle. The data from the cyclic stress–strain curves are presented in Table 4.

**Table 4:** Cyclic stress–strain curves data for TPU and TPU/CNT membranes.

Sample	Cycle	Strain end point (%)	Permanent set (%)	Elastic recovery (%)	ER/SEP ratio (%)
Unidirectional pure TPU	1	$105\pm 8$	16 $\pm 2$	89 $\pm 9$	79$\pm 2$
	20	129 $\pm 11$	29 $\pm 5$	100 $\pm 10$	78$\pm 3$
Unidirectional TPU/CNT	1	56 $\pm 4$	$4 \pm 2$	52 $\pm 2$	93 $\pm 2$
	20	$66 \pm 6$	$7 \pm 4$	59 $\pm 2$	89$\pm 24$
Random pure TPU	1	169 $\pm 5$	19 $\pm 2$	151 $\pm 3$	89 $\pm 2$
	20	$208\pm 3$	41 $\pm 4$	167 $\pm 2$	80$\pm 2$
Random TPU/CNT	1	80 $\pm 3$	9 $\pm 1$	71 $\pm 1$	89 $\pm 2$
	20	96 $\pm 2$	18 $\pm 3$	78 $\pm 1$	81$\pm 3$

In the first cycle, the randomly oriented TPU membranes demonstrated the highest strain end point (SEP) values, indicating their ability to withstand greater deformation during cyclic loading. Conversely, the addition of CNT into TPU random fibers shows a reduction in both the stain end point and the elastic recovery (ER) by ~ 53%, suggesting a reduction in their recovery capabilities. These findings align with the results obtained from the tensile tests conducted. Likewise, the aligned membranes follow a similar trend, although with slightly lower values, with a ~ 47% reduction in SEP, and a ~ 42% reduction in ER for the TPU and TPU/CNT membranes.

Looking at the 20th cycles for each material and comparing them with the first cycles, it was observed that both the randomly aligned membranes showed an increase in both elastic recovery and strain end point. Specifically, the TPU randomly aligned membranes exhibited a 23% increase in SEP and a 10% increase in ER, while the TPU/CNT random membranes showed a 20% increase in strain end point and a 10% increase in elastic recovery values.

Similarly, comparing the 1st and 20th cycles for the aligned membranes, the TPU uniformly aligned membranes exhibited a 23% increase in SEP and a 12% increase in ER values. Moreover, the increase for the TPU/CNT unidirectional membranes was 18% and 13% in SEP and ER values, respectively.

These results indicate that unidirectional membranes have a higher capability of sustaining slightly higher strains and improved recovery capacities over the course of the 20 cycles. They also suggested that the presence of CNT in these membranes may have had a slight influence on their mechanical behavior, resulting in a somewhat lesser improvement in their recovery capacities compared to the pure TPU membranes.

To gain a better understanding of the elastic recovery behavior, the ER/SEP ratio, which represents the percentage of elastic recovery compared to the strain end point, was determined. The greatest ER/SEP ratios were found in TPU/CNT membranes with unidirectional alignment, suggesting the most effective elastic recovery relative to their strain capacity.

### Thermogravimetric analysis (TGA)

**Figure 10:**
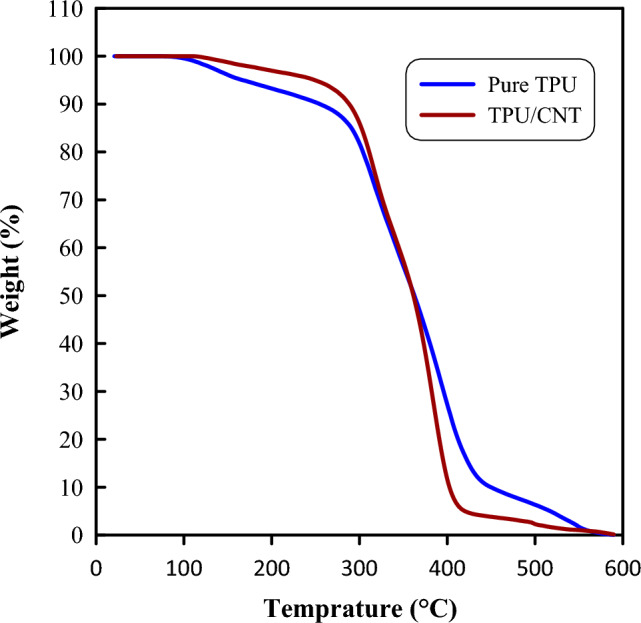
TGA curve for pure TPU and TPU/CNT membranes.

Thermogravimetric analysis (TGA) was performed to evaluate the thermostability of the fabricated TPU and TPU/CNT nanofibrous mats during thermal degradation. The TGA curves for pure TPU and TPU/CNT membranes are presented in Fig. 10. The thermal degradation for all the materials occurs in a single stage due to its compatibility with thermodynamic stability^65,66^. For the pure TPU nanofibrous mat, two clear degradation temperatures were observed, corresponding to the decomposition of the hard and soft segments. The hard segments had a first decomposition temperature of ~ 276 °C, whereas the soft segments had a second decomposition temperature of ~ 436 °C, because the amide bonds in the hard segments were simpler to break than the other bonds in the soft segments^67,68^.

The addition of CNT into TPU resulted in thermal degradation properties of the TPU/CNT nanofibrous mat that were significantly closer to those of pure TPU. Thus, the polymer structure of the TPU did not change after mixing, suggesting that the TPU/CNT nanocomposite has steady thermal degradation properties^67^. This finding is consistent with previous studies that have reported the compatibility and stability of TPU and CNT in a nanocomposite structure^69^. The results from the TGA analysis indicate that the TPU/CNT nanofibrous mats have promise for use in applications that require stable thermal properties^70^.

### Dynamic mechanical analysis (DMA)

DMA was selected for this research due to its high sensitivity to glass transition, which is estimated to be 100 times higher than differential scanning calorimetry (DSC) ^71^. The technique is also capable of detecting more localized transitions, such as side chain movements, that are not detectable by DSC. Additionally, DMA allows for the rapid scanning of a material's modulus and viscosity as a function of temperature, strain, or frequency^72^.

The DMA consists of two main components: the storage modulus (E′) and the loss modulus (E″). E′ is a measure of the energy stored in the material when it is deformed and is related to the material's ability to store energy and recover its original shape after deformation. E″ is a measure of the energy dissipated by the material as it undergoes deformation and is related to the material's ability to absorb energy and convert it into heat. The damping ratio (tan δ) is the ratio of the loss modulus to the storage modulus and is a measure of the material's ability to dissipate energy and its damping properties^73^.

The glass transition is a temperature range rather than a single point, and it is necessary to designate distinct locations within this transition zone in order to characterize it. There are three methods for determining the glass transition temperature in the context of DMA analysis (T_g_) ^74^. The first involves identifying T_g_ as the extrapolated onset of the sigmoidal decrease in the storage modulus, observed when transitioning from the hard, brittle region to the soft, rubbery region of the tested materials under specific parameters. Alternatively, T_g_ can also be determined by observing the peaks in the loss modulus and tan delta curves^73,75^. The T_g_ values obtained from these methods are presented in Table 5.

**Table 5:** Glass transition temperature for TPU and TPU/CNT mats.

Sample	T_g_ from onset of E′ (°C)	T_g_ from E′′ peak (°C)	T_g_ from tan δ peak (°C)
Unidirectional pure TPU	− 55.1	− 34.3	− 24.4
Unidirectional TPU/CNT	− 58.8	− 36.0	− 26.0
Random pure TPU	− 45.3	− 33.6	− 22.5
Random TPU/CNT	− 50.6	− 33.8	− 24.4

All four specimens’ glass transition ranged from  − 60 °C to  − 20 °C, which is the transition region for the mats. Due to the crosslinking network in the TPU chains, the composites acted solid and rigid below the transition region before becoing soft and rubbery when the temperature passed through the transition region^76^.

**Figure 11:**
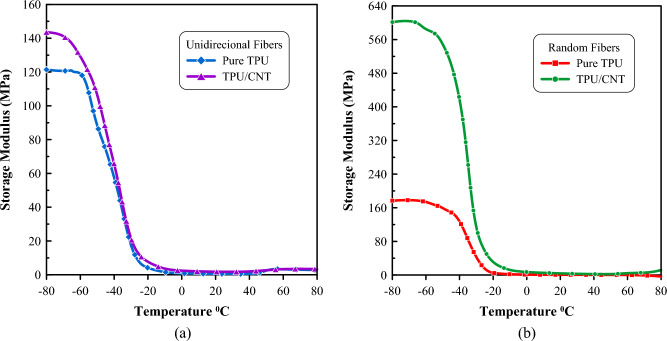
Temperature dependence of storage modulus as measured by DMA for (**a**) unidirectional pure TPU and TPU/CNT membranes, and (**b**) randomly oriented pure TPU and TPU/CNT membranes.

In Fig. 11, E′ of both TPU and TPU/CNT decreases progressively with increasing temperature, then drops abruptly at the glass transition (T_g_)^77^. This decrease in storage modulus refers to the thermal transition from a glassy to a rubbery phase (transition region)^78^. The storage modulus (E′) was measured over a temperature range from  − 80 °C to 80 °C. The addition of CNT to both randomly oriented and unidirectionally aligned TPU mats resulted in significantly higher storage modulus compared to the pure TPU mats. This increase can be attributed to the enhanced interfacial adhesion between the CNT and the TPU matrix, facilitating improved load transfer and increased stiffness^79^. These findings align with similar studies conducted by other researchers^80–82^, further supporting the positive impact of CNT incorporation on the thermomechanical properties of TPU nanofibrous mats.

Furthermore, it is evident in Fig. 11 that the storage modulus of randomly oriented membranes is significantly higher than that of their unidirectionally aligned counterparts. This observed behavior can be attributed to the increased fiber–fiber interactions and entanglements within the randomly oriented membranes. These interactions enhance the load-bearing capabilities of the membrane, resulting in a higher storage modulus^79^. On the other hand, the fibers in the unidirectionally aligned nanofibrous membranes are oriented in a specific direction, allowing for easier deformation and reduced fiber–fiber interactions^79^.

**Figure 12:**
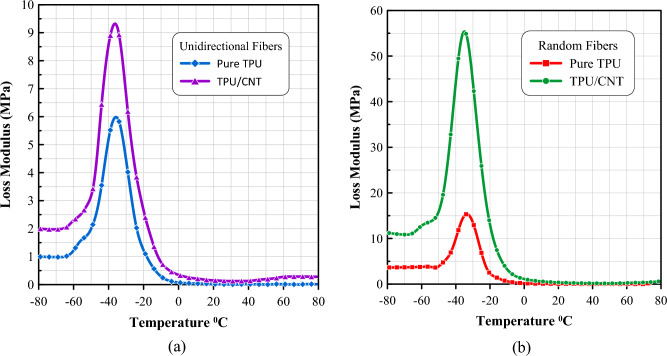
Temperature dependence of loss modulus as measured by DMA for (**a**) unidirectional pure TPU and TPU/CNT membranes, and (**b**) randomly oriented pure TPU and TPU/CNT membranes.

Similarly, the loss modulus follows the same behavior, with the TPU/CNT mats having a higher peak than that of the pure TPU mats, and the random membranes having a much higher peak than the unidirectionally oriented ones as shown in Fig. 12. This behavior can be explained by the fact that the random orientation of the fibers creates more disordered and less efficient load transfer mechanisms, leading to more energy dissipation.

**Figure 13:**
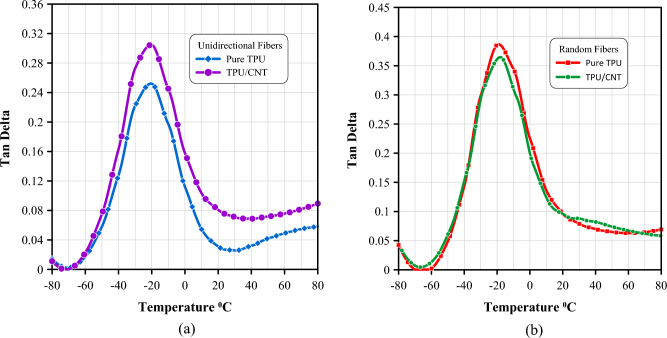
Temperature dependence of tan delta as measured by DMA for (a) unidirectional pure TPU and TPU/CNT membranes, and (b) randomly oriented pure TPU and TPU/CNT membranes.

The damping ratio (tan δ) for the randomly oriented materials is higher than that of the aligned fibers membranes, as is displayed in Fig. 13, as the random orientation of the fibers leads to more energy dissipation and lower energy storage^76,78^. Overall, the DMA results suggest that the fiber orientation has a significant effect on the viscoelastic properties of the material, and the addition of CNT can significantly modify the viscoelastic characteristics of TPU, making it suitable for different applications that require specific viscoelastic properties. The data of temperature dependence of storage modulus, loss modulus and tan delta for all mats is summarized in Table 6.

**Table 6:** DMA curves data for TPU and TPU/CNT membranes.

Sample	Storage modulus (MPa) at − 60°C		Storage modulus (MPa) at − 20°C		Peak height of loss modulus (MPa)		Peak height of Tan δ	
	E′_−60_ $\pm SD$	C.V%	$\mathrm{E^{\prime}}$_–20_ $\pm SD$	C.V%	E′′$\pm SD$	C.V%	$\mathrm{Tan \delta }\pm SD$	C.V%
Unidirectional pure TPU	118.8 $\pm 7$	6	4.6 $\pm 0.8$	18	6.4 $\pm 0.8$	12	0.3216 $\pm 0.03$	9
Unidirectional TPU/CNT	143.2 $\pm 15$	11	8.8 $\pm 1.3$	15	9.5 $\pm 1.3$	10	0.3504 $\pm 0.03$	9
Random pure TPU	179.9 $\pm 19$	10	4.7 $\pm 0.7$	14	15.68 $\pm 1.4$	9	0.4705 $\pm 0.04$	8
Random TPU/CNT	614.6 $\pm 55$	9	32.6 $\pm 3.5$	11	58.9 $\pm 9$	15	0.4397 $\pm 0.06$	16

**Figure 14:**
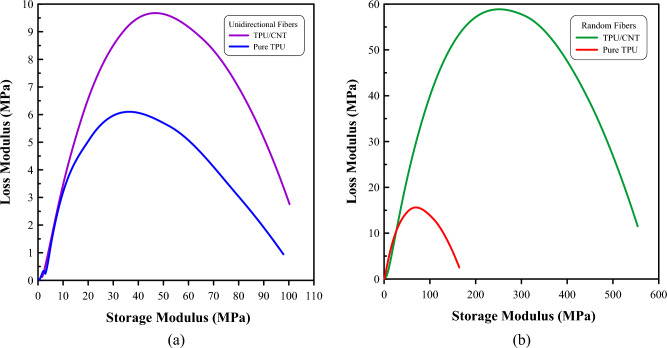
Cole–Cole plot for, (**a**) unidirectional pure TPU and TPU/CNT membranes, and (**b**) randomly oriented pure TPU and TPU/CNT membranes.

Figure 14 depicts the Cole–Cole plot, which showcases the correlation between storage modulus and loss modulus values. The plot is a valuable tool to interpret the state of homogeneity of blends and fillers dispersion within polymeric composite systems^83^. It has been established that the homogeneous polymeric materials exhibit nearly perfect semi-circular shapes, while other multiphase systems tend to display deviations towards elliptical curves^84–86^.Conversely, irregular, or imperfect arcs indicate blend heterogeneity or filler agglomeration^87^. In Fig. 14, both the pure TPU and TPU/CNT mats in both orientations exhibit similar elliptical shapes. This observation suggests a homogeneous distribution of CNT and enhanced adhesion between the components of the multiphase system^88,89^.

## Conclusions

In conclusion, in this comparative study, the impact of fiber alignment and muti-walled carbon nanotubes (MWCNTs) content on the properties of thermoplastic polyurethane (TPU) nanofibers was explored. The study revealed that the random and aligned nanofibers had distinct and highly uniform morphologies. The XRD and FTIR analyses provided valuable insights into the crystalline and chemical structures of the produced nanofibers and nanofibrous composites. The thermal stability of the nanofibers slightly improved with the addition of MWCNTs. The T_g_ of all materials lied between the temperature range of  − 60 °C to  − 20 °C.

The mechanical characteristics of the mats were highly affected by the orientation of the fibers, where the nanofibers with aligned orientation exhibited superior mechanical properties compared to those with random orientation. However, the presence of MWCNTs lead to an increase in tensile strength by 33.6%, increased the Young's modulus by 7.6%, and lowered the maximum strain by 33.6% in the aligned orientation. While, for the random fibrous mats, the tensile strength increased by only 56.4% the maximum strain was reduced by 48.8%, and the Young's modulus increased by 92.3%.

The study also showed that fiber alignment significantly affects the viscoelastic properties of the nanofibers. The DMA results showed that the TPU/CNT nanofibers exhibited higher storage modulus and loss modulus compared to the TPU nanofibers. Furthermore, it showed that the membranes with random orientation have higher storage and loss modulus than the aligned membranes, indicating more energy dissipation. The study provides valuable insights that can be used to design and optimize nanofibrous materials for various advanced applications, including tissue engineering, sensors, and wearable electronics (Supplementary Material).

### Supplementary Information

Supplementary Figures.

## Data Availability

The datasets used and analyzed during the current study are available from the corresponding author on request.
