# Supplementary material for: Thermo-mechanical characterization of electrospun polyurethane/carbon-nanotubes nanofibers: a comparative study
Source: Sci Rep. 2023 Oct 13;13:17368. doi: 10.1038/s41598-023-44020-x (PMC10575888; doi:10.1038/s41598-023-44020-x)
Supplement: Supplementary file 1 — Supplementary Figures. [file 41598_2023_44020_MOESM1_ESM.docx]

**Supporting Information**

**Thermo-Mechanical Characterization of Electrospun Polyurethane /Carbon-Nanotubes Nanofibers: A Comparative Study**

A. Shaker*, Amira T. Khedewy, Mohamed A. Hassan and Marwa A. Abd El-Baky

*Mechanical Design and Production Engineering Department, Zagazig University, Zagazig 44519, Egypt.*


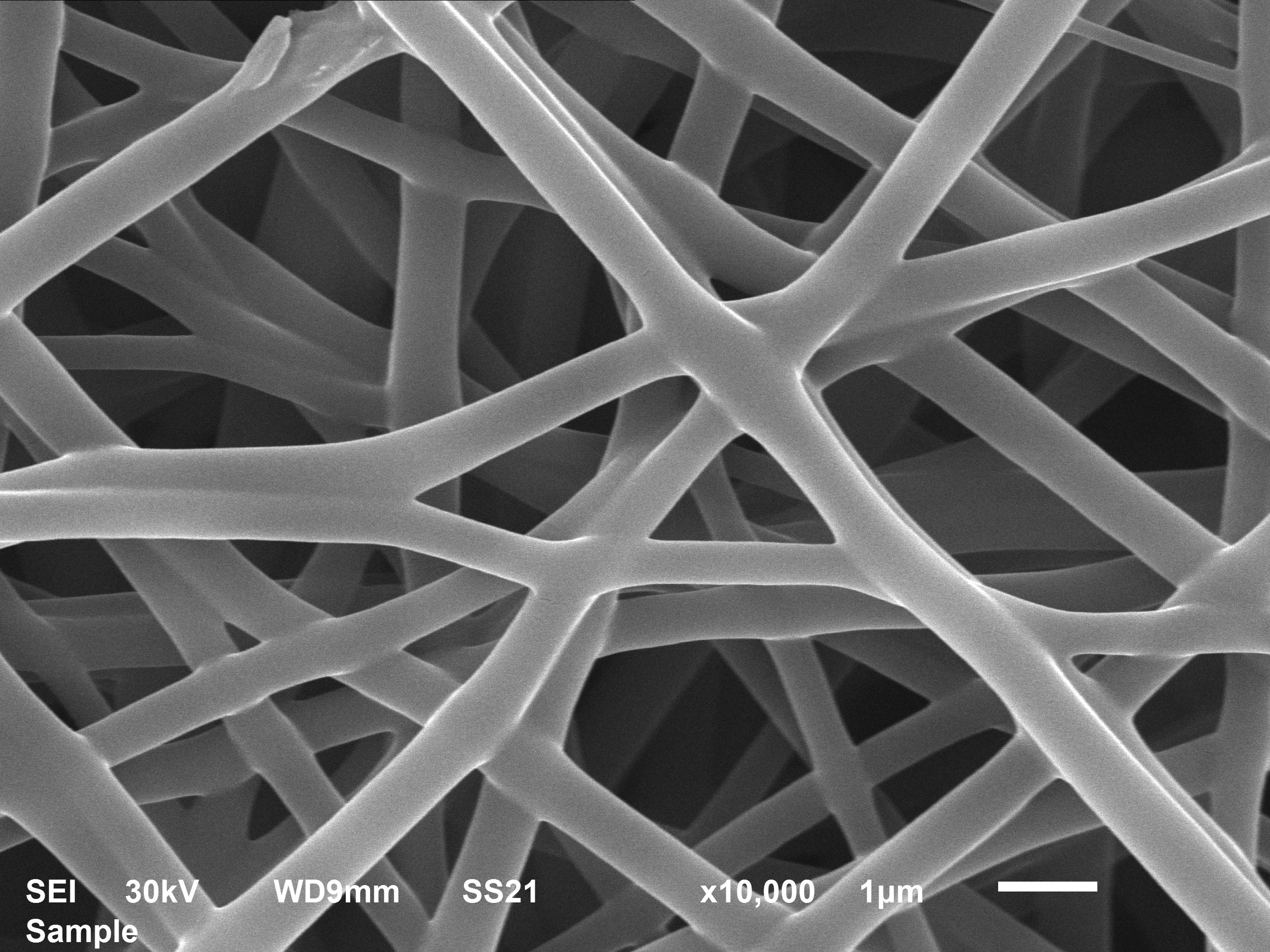


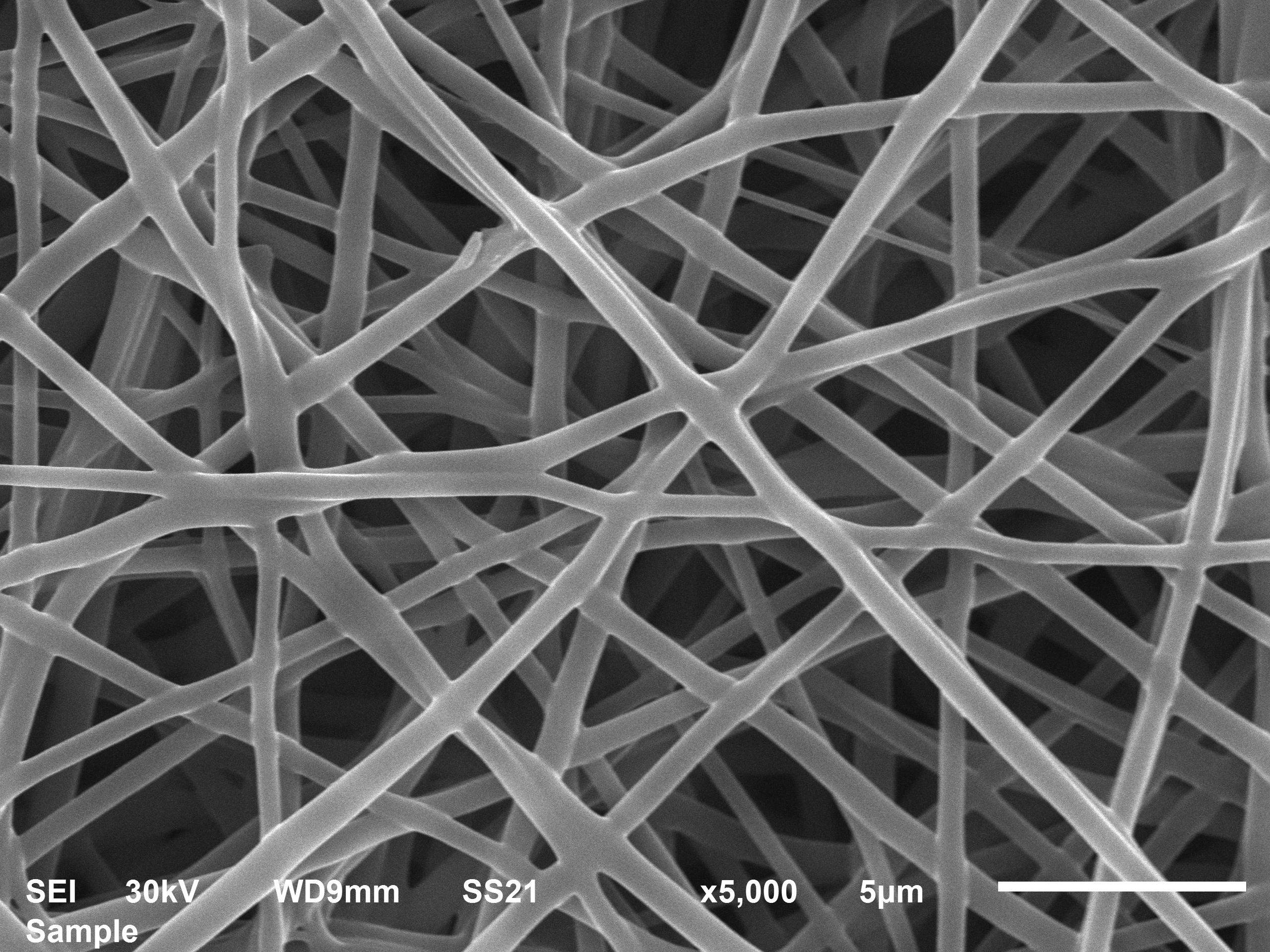


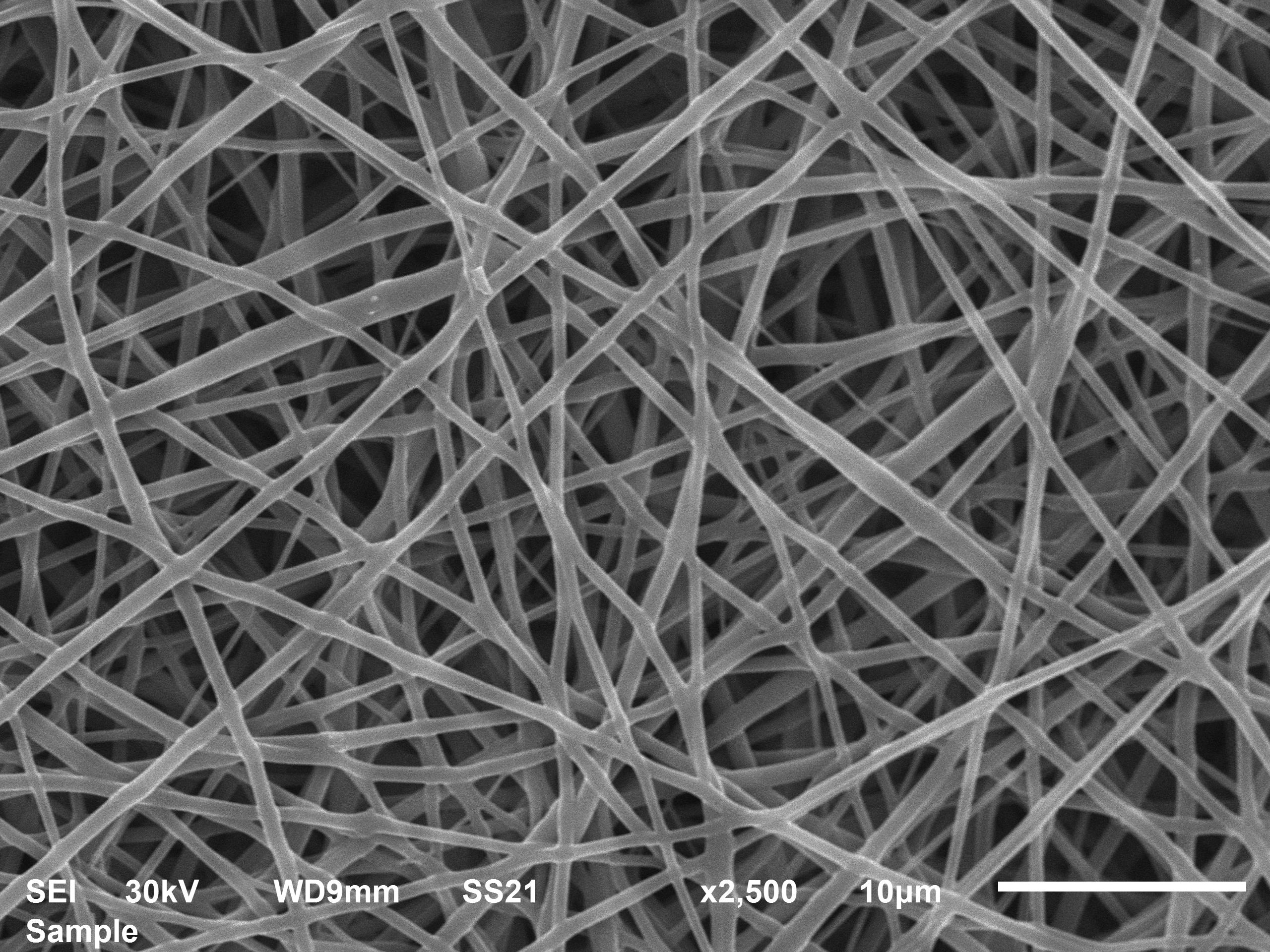


(a)


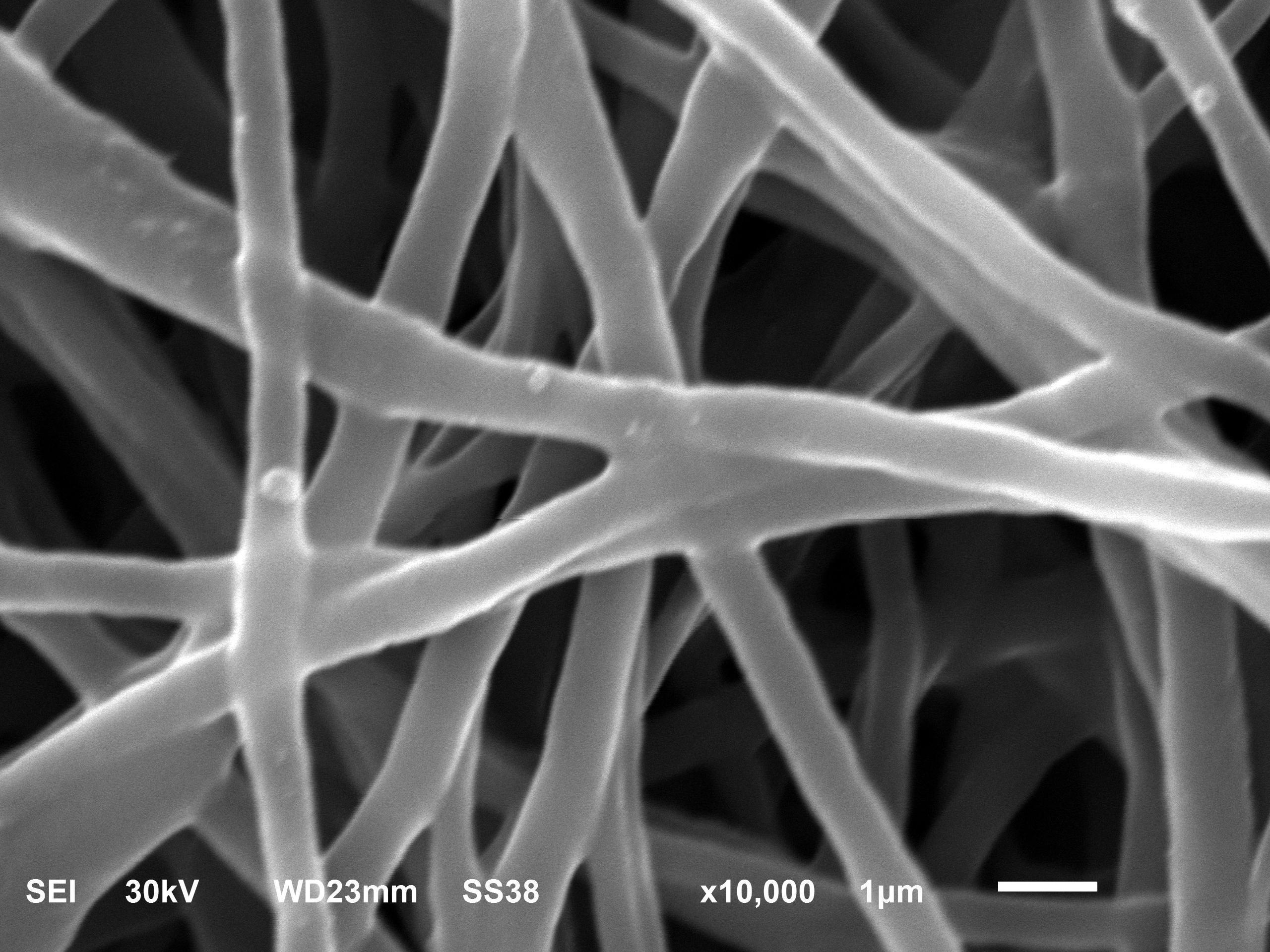


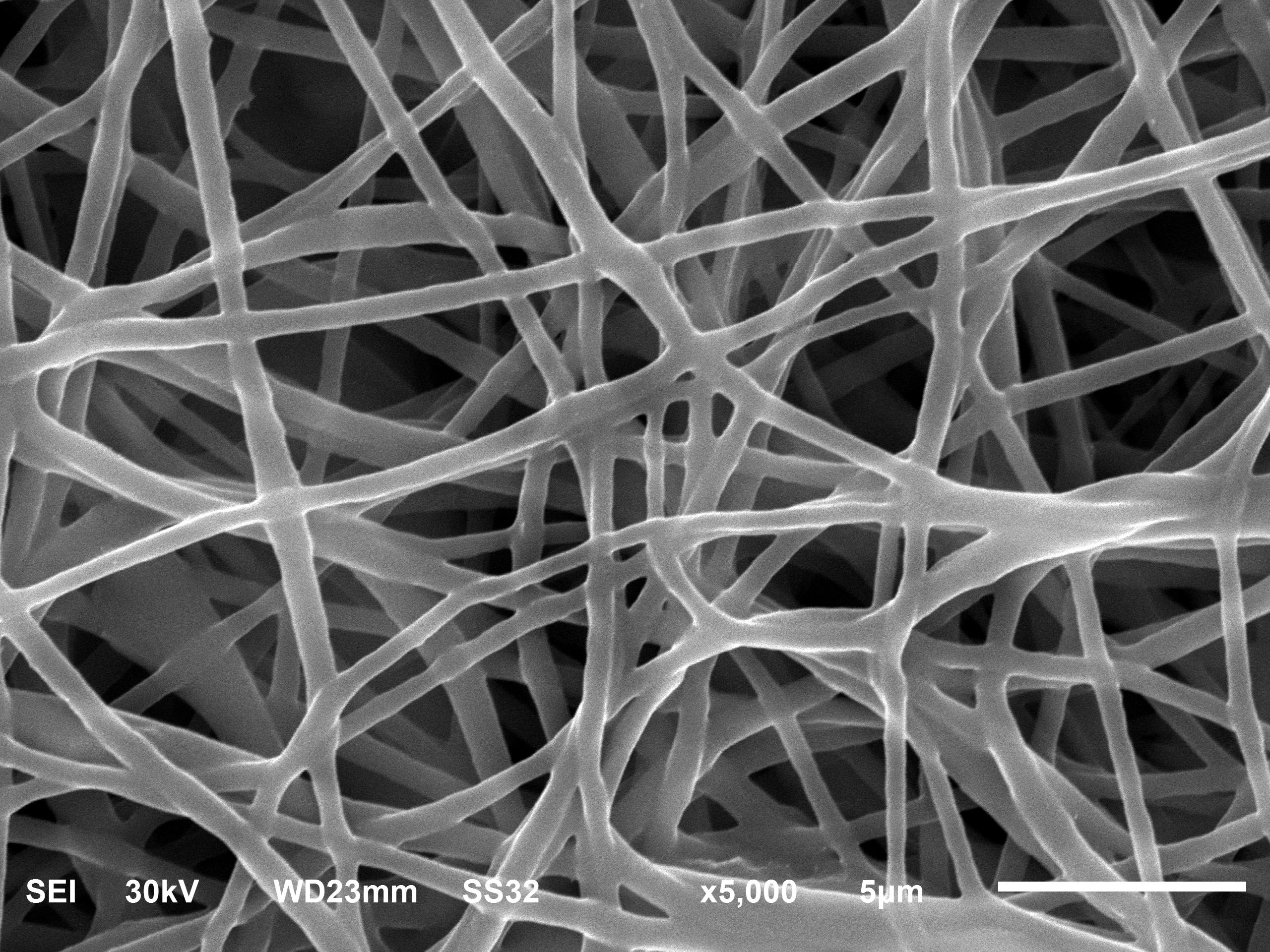


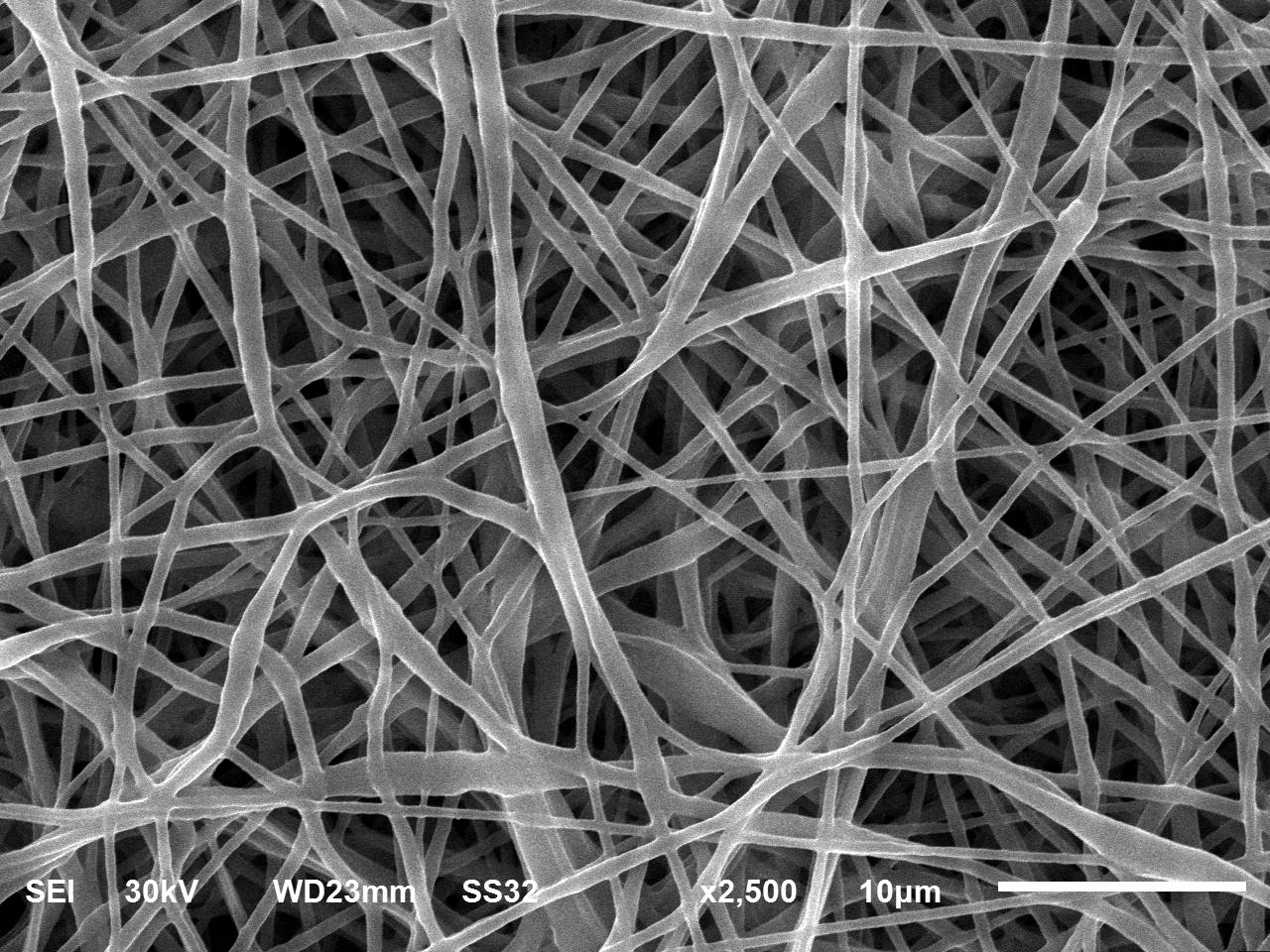


(b)

**Figure S1.** SEM images used in fiber diameter measurements of randomly oriented (a) pure TPU and (b) TPU/CNT membranes.


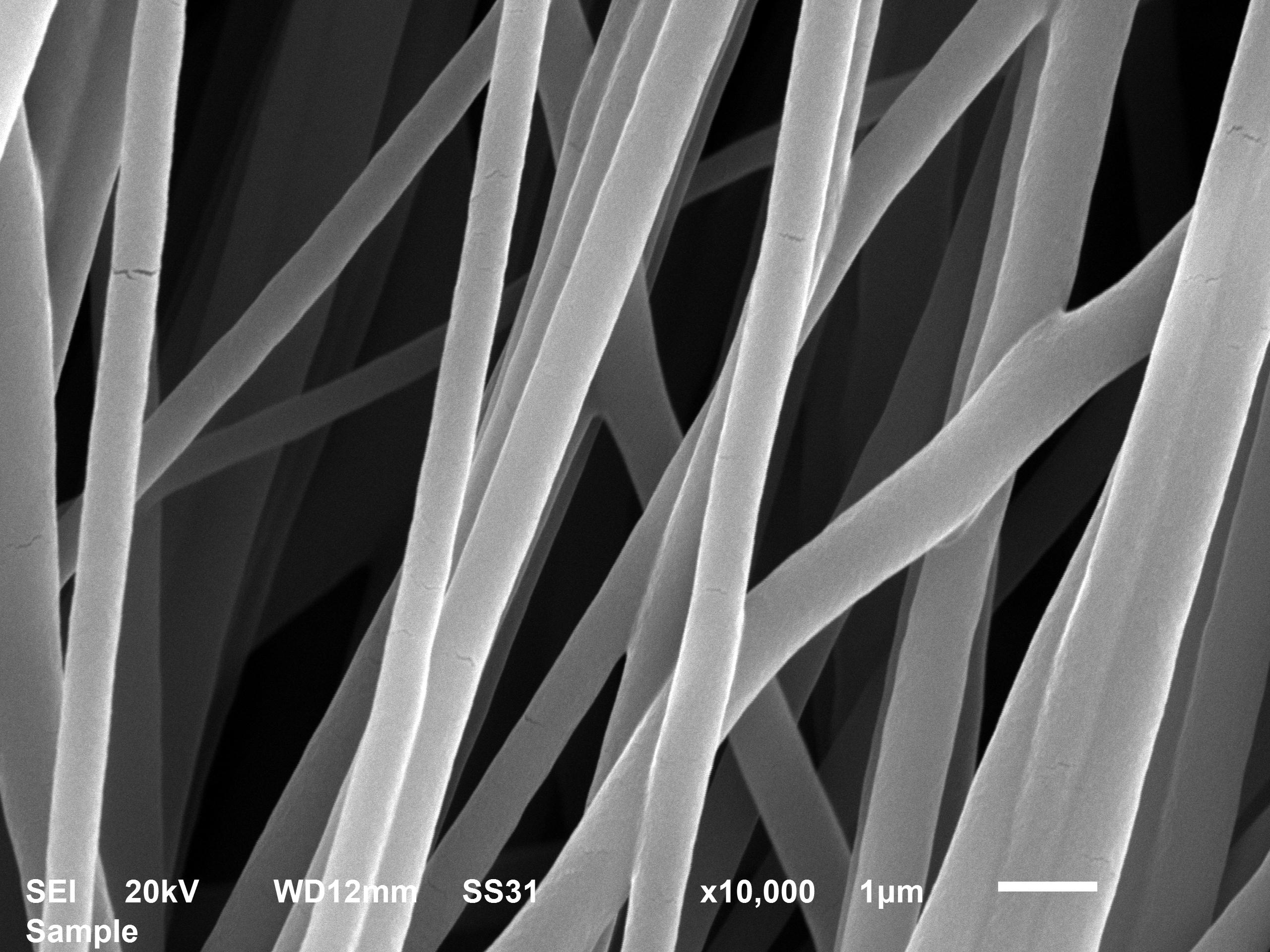


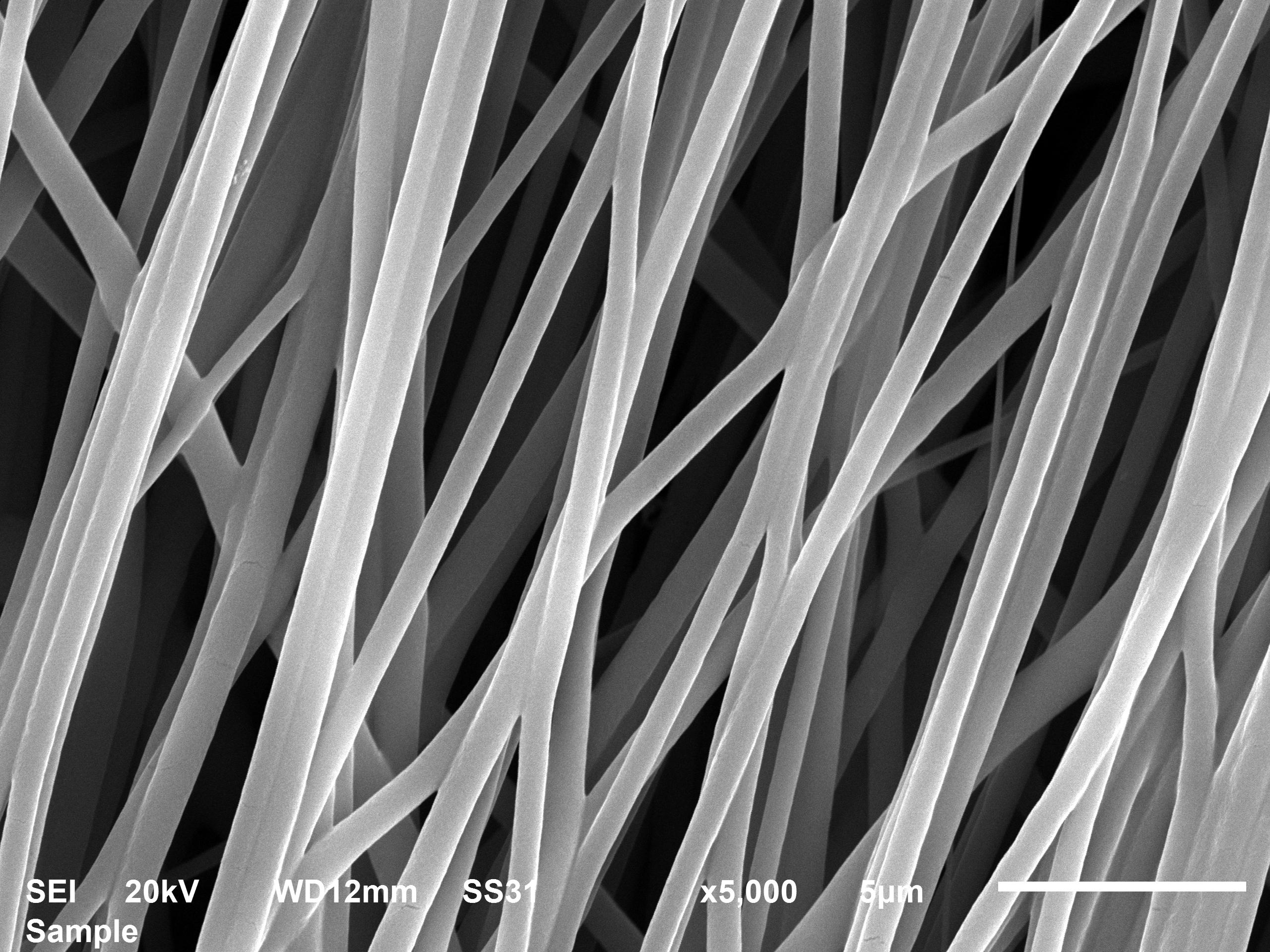


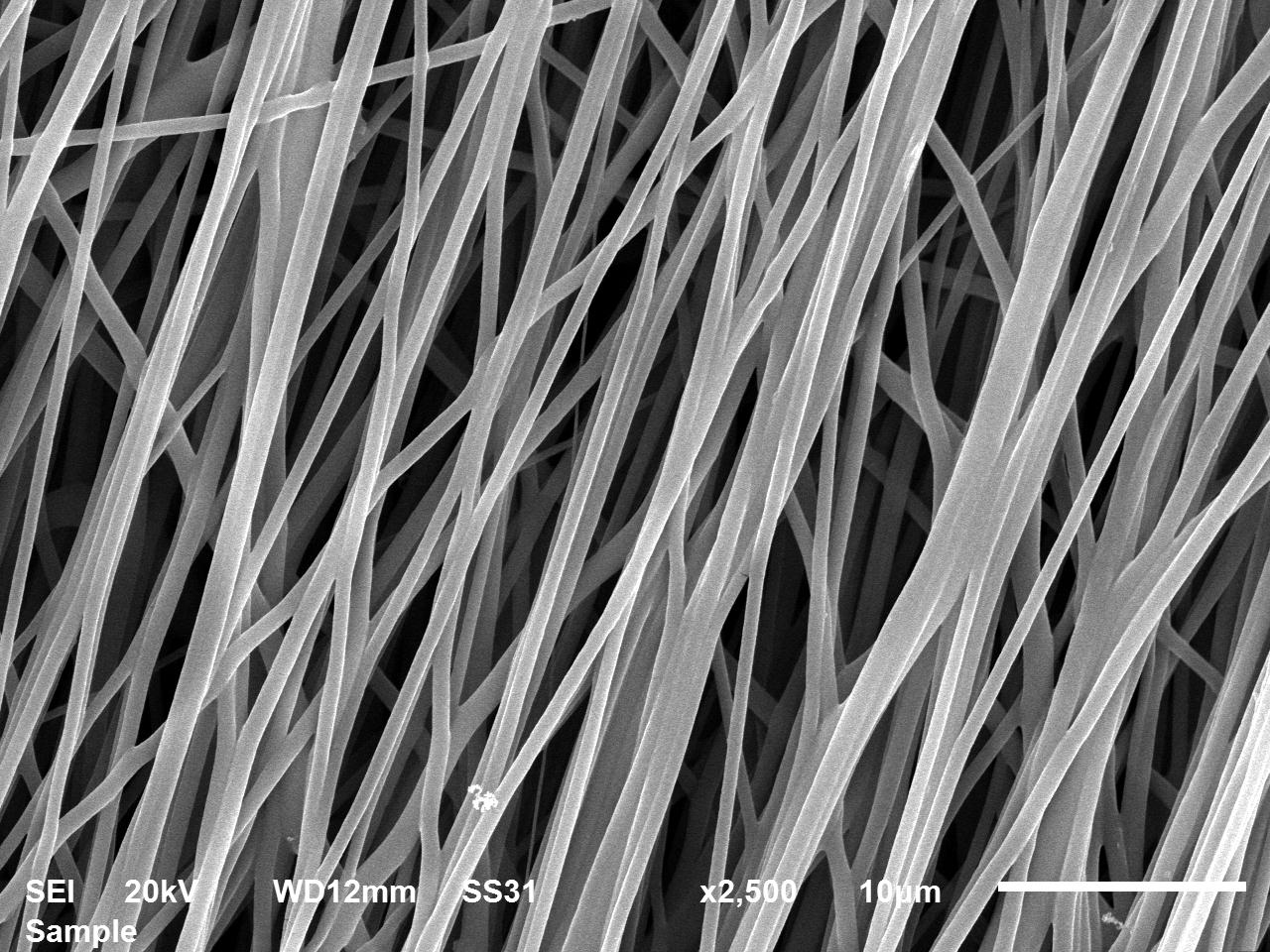


(a)


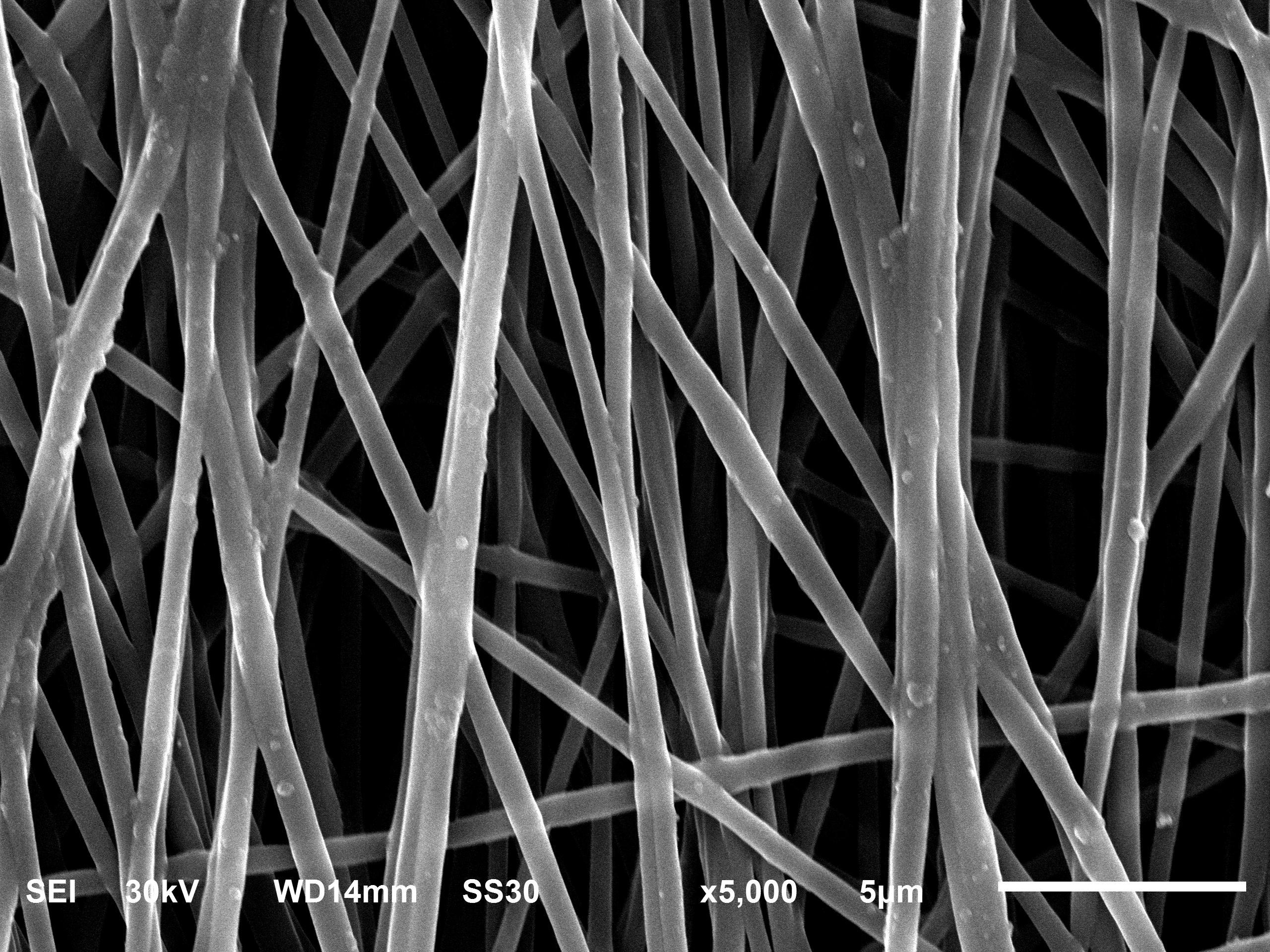


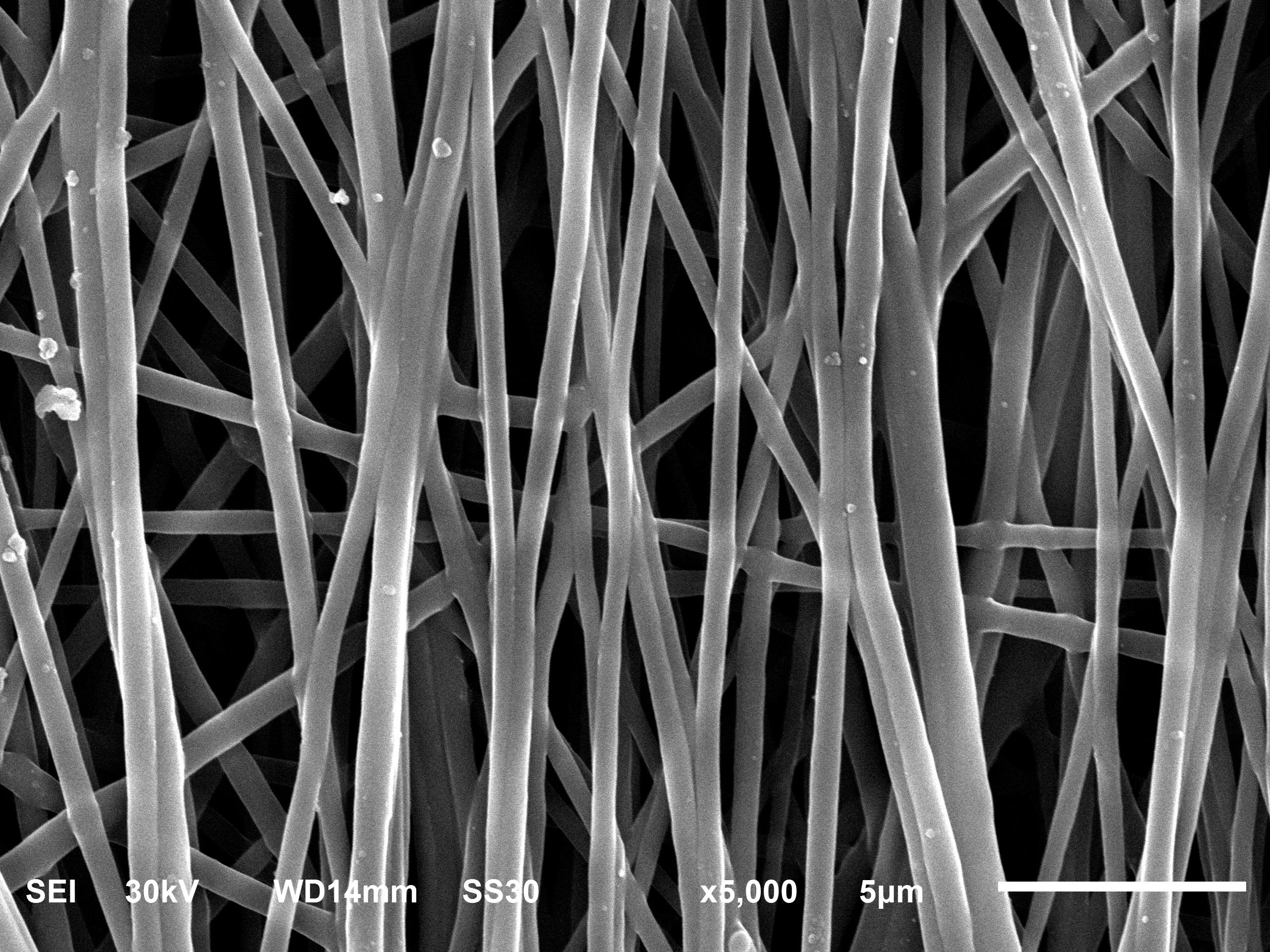


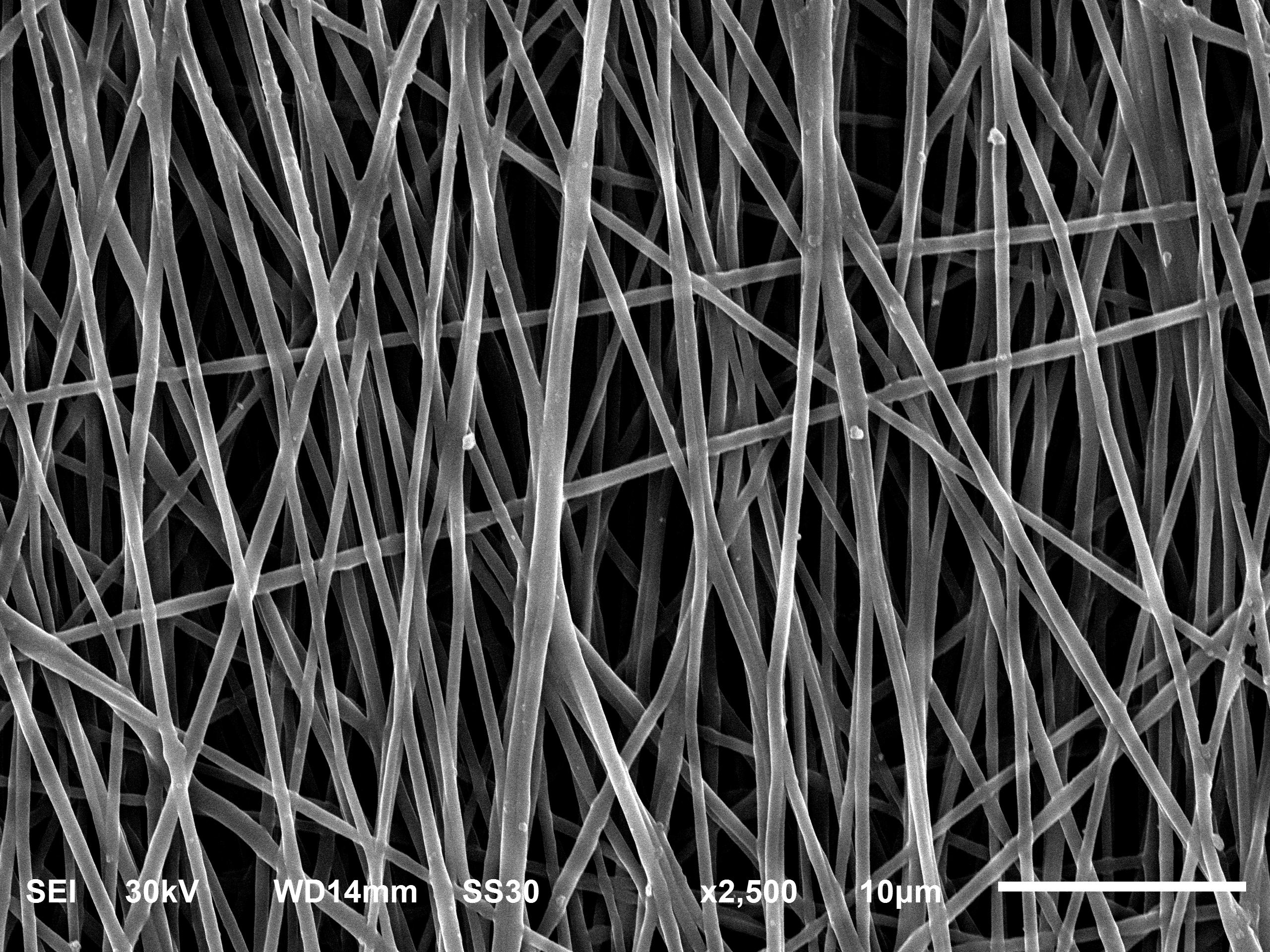


(b)

**Figure S2.** SEM images used in fiber diameter measurements of unidirectionally aligned (a) pure TPU and (b) TPU/CNT membranes.

(c)

(d)

**Figure S3.** Stress-strain curves for all repetitions for (a) unidirectional pure TPU, and (b) unidirectional TPU/CNT membranes, (c) randomly oriented pure TPU, and (b) randomly oriented TPU/CNT membranes.

(a)

(b)

**Figure S4.** Temperature dependence of storage modulus as measured by DMA for all reparations, for (a) unidirectional pure TPU, and (b) unidirectional TPU/CNT membranes, (c) randomly oriented pure TPU, and (b) randomly oriented TPU/CNT membranes.

(c)

(d)

(a)

(b)

(a)

(b)

**Figure S5.** Temperature dependence of loss modulus, all repetitions (a) unidirectional pure TPU, and (b) unidirectional TPU/CNT membranes, (c) randomly oriented pure TPU, and (b) randomly oriented TPU/CNT membranes.

(c)

(d)

**Figure S6.** Temperature dependence of tan delta, all repetitions (a) unidirectional pure TPU, and (b) unidirectional TPU/CNT membranes, (c) randomly oriented pure TPU, and (b) randomly oriented TPU/CNT membranes.

(c)

(d)

(a)

(b)
